# Nanomedicine for Immunotherapy Targeting Hematological Malignancies: Current Approaches and Perspective

**DOI:** 10.3390/nano11112792

**Published:** 2021-10-21

**Authors:** Alessandro Allegra, Mario Di Gioacchino, Alessandro Tonacci, Claudia Petrarca, Sebastiano Gangemi

**Affiliations:** 1 Division of Hematology, Department of Human Pathology in Adulthood and Childhood “Gaetano Barresi”, University of Messina, 98125 Messina, Italy; 2 Center for Advanced Studies and Technology, G. D’Annunzio University, 66100 Chieti, Italy; claudia.petrarca@unich.it; 3 Institute for Clinical Immunotherapy and Advanced Biological Treatments, 65100 Pescara, Italy; 4 Clinical Physiology Institute, National Research Council of Italy (IFC-CNR), 56124 Pisa, Italy; atonacci@ifc.cnr.it; 5 Department of Medicine and Science of Ageing, G. D’Annunzio University, 66100 Chieti, Italy; 6 Department of Clinical and Experimental Medicine, Unit and School of Allergy and Clinical Immunology, University of Messina, 98122 Messina, Italy; gangemis@unime.it

**Keywords:** nanomedicine, nanoparticles, hematological malignancies, immunotherapy, immune system, tumor microenvironment, tumor vaccine, drug delivery

## Abstract

Conventional chemotherapy has partial therapeutic effects against hematological malignancies and is correlated with serious side effects and great risk of relapse. Recently, immunotherapeutic drugs have provided encouraging results in the treatment of hematological malignancies. Several immunotherapeutic antibodies and cell therapeutics are in dynamic development such as immune checkpoint blockades and CAR-T treatment. However, numerous problems restrain the therapeutic effectiveness of tumor immunotherapy as an insufficient anti-tumor immune response, the interference of an immune-suppressive bone marrow, or tumoral milieu with the discharge of immunosuppressive components, access of myeloid-derived suppressor cells, monocyte intrusion, macrophage modifications, all factors facilitating the tumor to escape the anti-cancer immune response, finally reducing the efficiency of the immunotherapy. Nanotechnology can be employed to overcome each of these aspects, therefore having the possibility to successfully produce anti-cancer immune responses. Here, we review recent findings on the use of biomaterial-based nanoparticles in hematological malignancies immunotherapy. In the future, a deeper understanding of tumor immunology and of the implications of nanomedicine will allow nanoparticles to revolutionize tumor immunotherapy, and nanomedicine approaches will reveal their great potential for clinical translation.

## 1. Introduction

### 1.1. General Considerations on Tumor Immunotherapy

Conventional chemotherapy and radiotherapy have insufficient therapeutic results against tumors, these approaches being correlated to the onset of serious side effects and great risk of relapse. Recently, immunotherapeutic drugs have produced favorable effects in clinical tumor management and have then caught the attention of clinicians [1,2,3].

Tumor immunotherapy enhances the capacity of the immune system to identify and destroy tumor cells, which allows long-lasting remission in tumor subjects. Several immunotherapeutic antibodies and cell therapeutics are in continuous development [4,5]. In particular, immune checkpoint blockades have been created employing antibodies against cytotoxic T-lymphocyte antigen 4 (CTLA-4), programmed death-1 (PD-1), and programmed cell death ligand-1 (PD-L1), and some of these compounds have been authorized for the treatment of hematological malignancies. Moreover, in recent years, impressive advancement has been made in the area of adoptive cell therapy. The two fundamental approaches consist of endogenous non-engineered methods and genetically engineered T-cell methods. T-cell-centered immunotherapies include chimeric antigen receptor T (CAR-T) cells and bispecific T-cell engagers (BiTEs). CAR-T cells are autologous T cells achieved from patients and are genetically engineered to have an antibody single chain variable fragment (scFv) to identify and destroy tumor cells [6,7].

BiTEs are tandem scFv fragments attached by flexible linkers with one scFv aiming at a T-cell definite target such as CD3, while the other aims at a tumor-correlated antigen, which allows the BiTEs to readdress the T cell to the tumor cell [8,9]. T-cell-centered immunotherapy has demonstrated encouraging clinical results in several hematologic malignancies, including lymphoma, leukemia, multiple myeloma (MM), and Waldenstrom macroglobulinemia [10,11,12].

Tumor immunotherapy can support immune-mediated elimination of tumors by promoting the host’s immune system and eradicating tumor cells via a tumor–immunity cycle [13,14]. When tumor cells are destroyed via programmed cell death or necrosis, tumor antigens are collected by antigen-presenting cells (APCs), such as dendritic cells, and displayed on a major histocompatibility complex (MHC). Dendritic cells carrying the tumor antigens transfer to the lymph nodes, where they instruct immature T cells. Successively, stimulated tumor-specific cytotoxic T lymphocytes (TCLs) penetrate the tumor site and identify tumor cells, while effector T cells destroy tumor cells. Tumor immunotherapy strengthens these processes and has a relevant effect in provoking efficacious anti-tumor consequences [15]. Therefore, it causes an unprecedented therapeutic action, ensuring long-term survival in advanced-stage patients [16,17,18].

However, several difficulties limit the therapeutic effectiveness of the immunotherapy of hematological malignancies.

Two different elements appear to be fundamental for efficacious tumor immunotherapy. Tumor antigens must be efficiently transported to immune cells, especially APCs. Second, the immune-suppressive tumor microenvironment (TME) must be modified to respond to the anti-tumor immune-therapeutics. However, other elements, such as the production of immunosuppressive humoral factors, including monocyte chemoattractant protein-1 and bombesin that stimulate the infiltration of monocytes into the TME, infiltration of myeloid-derived suppressor cells (MDSCs), and macrophage modifications, allow the tumor to escape the immune response, reducing the effectiveness of the immunotherapy [19,20,21,22,23,24,25].

Moreover, other experimental justifications for immune resistance have been reported. Certainly, tumors can be theoretically differentiated into immune active versus immune silent tumors according to the expression of a specific group of genes called the *immunologic constant of rejection* expressing the tumor immune surveillance within the *TME* [26,27,28]. Galon et al. have reported that the two different types of tumors are linked to a different functional pattern of cytotoxic cells, memory T cells, Th1 cells, and interferon-gamma (IFN-γ) signatures and are associated with a different survival and a different recurrence rate [29,30].

Furthermore, several complications correlated with the employment of immunotherapy have been reported. These collaterals effects, commonly referred to as “immune-related adverse events”, are thought to be an inflammatory answer provoked by diverse elements. These include increased T-cell effect against antigens that are also present in healthy cells. This condition, with T cells identifying antigens equally present on tumor cells and in normal tissues, may partially be similar to paraneoplastic syndromes. Moreover, immunotherapy may also cause an increased concentration of pre-existing autoantibodies, which successively may identify and target antigens present on normal cells. Increased concentrations of pro-inflammatory cytokines may also have an essential effect in the onset of immune-related toxicities. Furthermore, stimulation of the complement system may also cause inflammation. Among the tissues, which are commonly altered by immunotherapy, are the pituitary gland and thyroid, skin, liver, colon, lung, kidney, joints muscles, and nervous system [31].

Neurological complications correlated to the use of immunotherapeutic drugs have been recognized. Immunotherapy may provoke different neurological alterations essentially by changes of the peripheral nervous system’s integrity. These include neuropathies, myopathies, like myasthenic syndromes, and radiculopathies. Collateral effects implicating the central nervous system are less common but may cause severe clinical symptoms. The use of chimeric antigen-receptor T cells is frequently associated with neurological complications, including encephalopathy and seizures, which require urgent action and adequate therapeutic measures [32].

Together, these data highlight the significance of overwhelming the limits of tumor immunotherapies. However, all the factors might be subjected to intervention for an optimization of the therapeutic results. Nanotechnology can be employed for each of these limiting factors, and therefore has the possibility to effectively stimulate anti-tumor immune responses. Here, we review recent trends in the use of biomaterial-based nanoparticles in immunotherapy of hematological malignancies.

### 1.2. Nanotechnology and Tumor Immunotherapy

Nanomedicines are small, sub-micron sized particles in the size range of 20–200 nm [33]. The first use of nanoparticles in tumor therapy was to promote the transport of anti-neoplastic drugs. In fact, the biocompatible and biodegradable feature of nanoparticles makes them appealing vehicles to enhance drug delivery. However, nanoparticles improve the actions of immunotherapy of hematological neoplasms with different mechanisms [34] (—www.ClinicalTrials.gov, accessed on 5 September 2021).

Nanoparticle delivery of chemotherapeutics, small molecules, drug inhibitors, and other cytotoxic molecules has been employed to increase biodistribution and tolerability with respect to free drugs. Combining targeting ligands to the nanoparticle can possibly enhance tumor cell localization and effectiveness with respect to non-targeting nanoparticles [35,36,37,38]. Their small size allows them to transit and accrue in the tumoral tissue passively via an increased permeability and retention (EPR) effect. Moreover, the specificity to the target cell can be increased by surface change of nanoparticles. In fact, the surface of nanoparticles can be simply changed, allowing the possibility to execute several chemical reactions.

**Figure 1:**
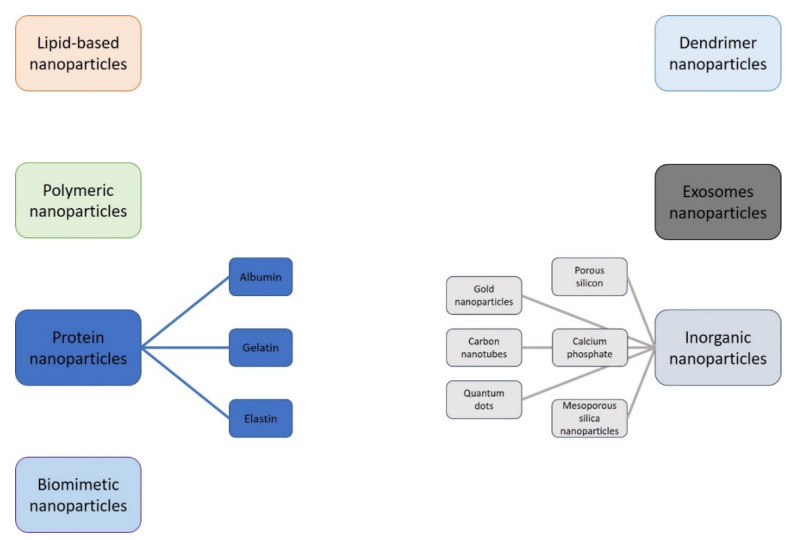
Nanostructures employed in nanomedicine.

Different nanostructures with diverse dimensions, forms, compositions, and functions have been implemented [39,40,41], and several nanodrugs have been authorized by the FDA for clinical use [42] (Figure 1).

Generally, nanoparticles are divided into organic and inorganic nanoparticles. Organic nanoparticles, such as polymer-, lipid- and carbon-based nanoparticles, and inorganic nanoparticles, like gold and magnetic nanoparticles, have been broadly explored for possible clinical purposes. In particular, multifunctional synthetic polymers and lipid-based nanoparticles such as poly (lactide-co-glycolide) (PLGA) nanoparticles, liposomes, melanoidins, and dendrimers generated interest as an abiotic protein affinity reagent for therapeutic use, cell engineering, and drug delivery carrier [43].

For instance, liposomes have been thought to be promising nanoparticles for drug delivery since their hydrophobic lipid layer and hydrophilic interior allows liposomes to carry both hydrophilic and hydrophobic drugs [44]. Dendrimers based on a unique tree-like branching architecture [45,46] can have several functional groups in the external area that can be tailored via chemical change to generate an ideal model for drug delivery. Melanoidins, constructed by the Maillard reaction [47], are biocompatible, biodegradable, excretable, and have been employed for biomedical photonic purposes [48]. Carbon-based nanoparticles have unique electrical, thermal, and chemical characteristics. Compounds such as graphene oxides, carbon nanotubes, and carbon nanodots have been efficaciously used for imaging, drug delivery, and treatment of several diseases [49]. Carbon nanotubes, which are made of graphene layers rolled up into cylinders, have drug delivery capability and absorbance, making them appropriate for biosensing and bioimaging purpose, while graphene oxides attained by chemical exfoliation of oxidized graphite are easy to functionalize due to their extremely oxidized structures with several surface functional groups. Carbon nanodots are spherical nanoparticles constituted of carbon and oxygen with 1–10 nm size, which have exceptional biocompatibility and photo-responsiveness.

Gold nanoparticles are biologically inactive and demonstrate biocompatibility for different biomedical objectives [50]. They can be fabricated in diverse morphologies, such as nanorods, nanospheres, nanocages, and nanoshells, according to their usages. Gold nanoparticles have been efficaciously used in photothermal and photodynamic therapy, photoacoustic tomography, and drug delivery [51,52].

The most widely experimented nanomedicine structures are lipid-based nanoparticles and polymeric nanoparticles, but other possible configurations include protein nanoparticles (albumin, gelatin, elastin), inorganic nanoparticles (gold nanoparticles, carbon nanotubes, quantum dots, calcium phosphate, porous silicon, and mesoporous silica nanoparticles), dendrimer nanoparticles, exosomes nanoparticles, and biomimetic nanoparticles [53,54,55,56,57,58,59,60].

However, the application of nanotechnology to tumor immunotherapy presents further advantages over enhanced drug delivery and could drastically improve the same effectiveness of immunotherapy and modify the immunosuppressive pathways by aiming at immune cells [61,62,63] and interfering in multiple phases of immunotherapy to potentiate anti-cancer immunity [64,65].

A probable further mechanism of action for the nanoparticles includes their effects on dendritic cells (DCs). DCs resulting from BM are the most effective APCs [66]. This could be demonstrated in blood, but when stimulated, they pass to lymph nodes where they interrelate with T cells. They identify endogenous and exogenous proteins, decomposing them into smaller portions, and submitting them on the cell surface to naive T cells, thus starting and regulating adaptive immunity [67,68,69].

Nanoparticles linked to specific ligands could aim at DCs and control their stimulation and maturation. Indeed, nanoparticles specific to Fc receptors, CD40, CD11c, CD205, or mannose receptors on DCs have been evaluated and all provoked an increased immune response with respect to non-specific delivery [70,71,72]. To establish which cell surface molecule represents the best target, Cruz et al. generated pegylated poly (lactic-co-glycolic acid) (PLGA) nanoparticles loaded with ovalbumin and Toll-like receptor (TLR) 3 and 7 ligands [73]. Decorated with specialized antibodies, these nanoparticles can affect various cell surface molecules on DCs. Nanoparticles aimed to CD40 attained the greatest binding and the highest generation of some cytokines such as interleukin-12 (IL-12) in vitro.

Administration of adjuvant via nanoparticles might be an alternative method to increase the immune response against the tumor. An adjuvant is a compound that increases immunogenicity, which is sometimes deficient in tumor antigens when presented alone. Adjuvants are similar to compounds induced by infectious pathogens and identified by pattern recognition receptors (PRRs) [74], and approaches have been implemented to efficiently transport adjuvants into APCs employing nanoparticles as it has an essential role in causing antigen-specific T-cell responses [75,76,77,78].

Moreover, nanoparticles could transform the immunosuppressive environment by targeting other components of TME.

The fast proliferation of tumor cells leads to hypoxia, which provokes immunosuppression in TME by gathering immunosuppressive cells comprising Tregs and MDSCs and producing immunosuppressive factors, such as vascular endothelial growth factor (VEGF) and transforming growth factor β (TGF-β). Such components block the action of DCs, transform macrophages to the pro-tumorigenic M2 phenotype, and cause anomalous fibrosis. Nanoparticles with specialized designs can affect these elements in TME and change the immunosuppressive TME to an immunosupportive condition, thus enhancing the efficacy of immunotherapy [79].

Finally, nanoparticles have been employed as vehicles for transporting tumor antigens to lymph nodes [80]. In this setting, nanoparticles own two relevant advantages: they can defend tumor antigens from degradative enzymes and support selective transport to the lymph nodes. After delivery, nanoparticles containing tumor antigens are efficiently internalized into APCs [81].

Based on what is told in the next part of our review, we will try to analyze literature data about the possibility of combining the immunotherapeutic treatment of hematological neoplasms with the use of nanotechnologies (Table 1).

## 2. Nanoparticles and Hematological Malignancies

### 2.1. Acute Myeloid Leukemia

Acute myelogenous leukemia (AML) is the most life-threatening among hematological neoplasms. Several genetic drivers are implicated in AML and directly influence therapeutic responsiveness, relapse percentages, and chemoresistance [82].

Immunotherapy has been employed in AML and leukemia-niche myeloid cells are possible targets for this kind of treatment, with the efficacy of some types of immunotherapy, including anti-PD-1 therapy, being also reported in relapsed AML subjects [83].

**Figure 2:**
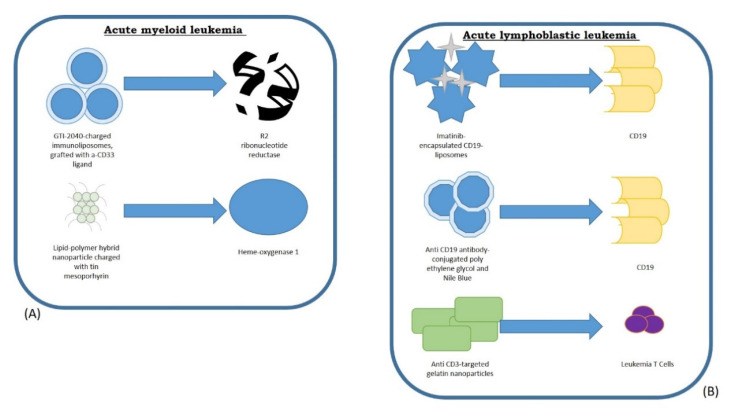
The use of nanoparticles permitted to recognize and aim at several targets that could be useful in acute myeloid leukemia (**A**) and (**B**) acute lymphoblastic leukemia.

The use of nanoparticles has allowed an increase in the effectiveness of conventional and immune therapy in subjects with AML and to recognize and aim at several targets that could increase the effects of immunotherapy on the BM milieu. For instance, heme oxygenase 1 (HO1) is a cytoprotective enzyme causing chemo-resistant AML cells and has been identified as an immune checkpoint molecule in AML microenvironments. To target this molecule, a lipid-polymer hybrid nanoparticle (hNP) was charged with tin mesoporphyrin (SnMP), an HO1-inhibitor, and transformed with an engineered antibody for leukemic cell-targeted delivery [84]. HO1-inhibiting T-hNP (T-hNP/SnMP) increased chemo-sensitivity in leukemia cells, while in a human-AML-bearing orthotopic animal model, intravenously administered T-hNP not only actively targeted leukemia cells, but passively targeted CD11b+ myeloid cells in the BM niche (Figure 2). The T-hNP/SnMP increased the chemo-therapeutic action of anti-leukemic drugs and enhanced the immune response by reprogramming BM myeloid cells [84].

Nanomedicines coupled with antibodies can aim at specific cell-surface receptors and transport drugs into cells [85]. GTI-2040 is an antisense oligonucleotide aiming at the small subunit R2 of ribonucleotide reductase [86,87]. It has been stated that CD33 is a membrane receptor present on AML progenitors lacking in normal BM stem cells. GTI-2040-charged immunoliposomes grafted with an anti-CD33 ligand have been produced and tested on AML cells and on experimental animal models [88]. Findings suggested that it remarkably reduced expression of R2 and reduced cell survival in AML cell lines. Different anti-CD33-incorporated multi-inhibitor-charged PLGA polymer NPs were also produced, and the simultaneous inhibition of critical kinases provoked synergistic effect against leukemic cells without targeting normal blood cells [89].

For the future, to boost the use of nanoparticles and immunotherapy, several platforms have been suggested that could simplify the possibility to induce an intense and long-lasting anti-leukemic clinical response. Employing a new nano artificial antigen-presenting cell planned to generate multi-antigen-specific T-cell products, a phase I/II clinical trial has been organized. NEXI-001 T-cell product will be administered to AML subjects with refractory/relapsed disease after getting an allogenic stem cell transplant (www.ClinicalTrials.gov, accessed on 5 September 2021 identifier: NCT04284228). Subjects recruited in this study will have AML tumor-specific CD8+ T cells generated from the HLA-matched stem cell donor peripheral blood mononuclear cells [90]. The main goals for this study include safety and tolerability. Six subjects will be enrolled in two different groups at increasing dosages within a 3 + 3 design. In the absence of any dosage-limiting toxicities, a dose expansion phase will follow, allowing for enrollment of up to 16–20 additional patients. All the patients within the study will be monitored for clinical effects, immunological actions, dose-limiting toxicities, and adverse events. A second trial is actually recruiting relapsed/refractory MM subjects that have received at least three previous lines of treatment (www.ClinicalTrials.gov, accessed on 5 September 2021 identifier: NCT04505813). Analogously, the objective of this trial is to assess safety and tolerability [90].

### 2.2. Acute Lymphoblastic Leukemia

Acute lymphoblastic leukemia (ALL) is a neoplasm characterized by the growth of malignant lymphoblasts of the B or T lineage, leading to an inhibition of proliferation of the normal blood cell lineages [91]. The clinical approach for the therapy of ALL is based on conventional chemotherapy containing highly toxic drugs. Generally, after chemotherapy, a consolidation treatment with immunotherapy (anti-CD20 antibodies or bispecific antibodies) and/or allogenic stem-cell transplantation is carried out [92].

Several experimentations have assessed the use of nanotechnologies for the treatment of this condition. Harata et al. settled an immunoliposome transporting anti-CD19 antibody (CD19-liposomes). The cytocidal action of imatinib-encapsulated CD19-liposomes (imatinib-CD19-liposomes) on Ph(+) ALL cell lines and primary leukemia cells from subjects with Ph(+) ALL was much greater than that of imatinib with or without control liposomes [93].

Tatar et al. suggested a new nanoparticle-based immunotherapeutic treatment against ALL involving an anti-CD19 antibody-conjugated, polyethylene glycol (PEG)-biocompatibilized, and Nile Blue (NB) Raman reporter-tagged gold nanoparticles of urchin-like shape (GNUs). Anti-CD19-PEG-NB-GNUs displayed greater cytotoxic action against CCRF-SB cells (B lymphoblast cell line) with respect to the free antibody by decreasing the overall viability to under 18% after seven days of therapy [94] (Figure 2).

Other attempts include a different anti-CD19-targeted block co-polymer nanoparticle efficaciously used to deliver chemotherapeutic agents to cells and to enhance the survival percentage of treated animals [95], anti-CD3-targeted gelatin nanoparticles that can specifically target T-cell leukemia cells [96], or poly (lactic-co-glycolic acid) –polyvinyl alcohol co-polymer nanoparticles with a wide payload of asparaginase [97]. Survival analysis demonstrated that animals receiving CD19-DOX-NPs survived considerably longer than those treated with saline solution. Throughout the survival study, authors controlled the physical activity of animals employing a computerized low-profile wireless running wheel that evaluated the agility of the treated animals. Notably, the animals treated with CD19-DOX-NPs presented higher agility with respect to the other groups during the treatment [95].

Smith et al. reported enhanced T-cell leukemia targeting of peptide amphiphile micelles based on a DNA aptamer [98]. Inorganic nanoparticles, such as gold manganese oxide hybrid nanoflowers linked to aptamer molecules, were employed as a targeting platform for leukemic T cells [99,100] and aptamer-tagged gold nanospheres were employed as a pH-dependent drug transport system against human ALL T-cells [99,100]. It was also displayed that hollow gold–silver nanospheres targeting the CD19 epitope were efficaciously internalized by B lymphoblasts by receptor facilitated endocytosis [101,102]. Finally, vincristine sulfate liposome injection (VSLI) is a sphingomyelin and cholesterol nanoparticle preparation of vincristine sulfate (VCR) that was constructed to overwhelm the dosing and pharmacokinetic limitations of conventional VCR. In fact, conversely to the fast clearance of non-liposomal VCR, VSLI remains in the plasma for a longer period. In a study, 65 adult subjects with relapsed/refractory ALL were administered VSLI once a week at a dose of 2.25 mg/m^2^ IV over 60 min, with no dose cap. VSLI administration was correlated with a dose-dependent peripheral neurotoxicity even if at dosages two to three times that of traditional VCR. VCR dose intensification with VSLI correlated with an increased possibility of overall response and a convincing tendency toward increased complete response in subjects with relapsed and/or refractory acute lymphoblastic leukemia [103].

In a different study, authors assessed high-dosage VSLI monotherapy in subjects with Philadelphia chromosome (Ph)-negative ALL that was multiply relapsed or refractory to reinduction. Sixty-five patients were treated in this phase II, multi-national trial. Intravenous VSLI 2.25 mg/m^2^, without dose capping, was infused once per week until response, progression, toxicity, or pursuit of HCT. The CR/CRi rate was 20% and overall response rate was 35%. Median complete response duration was 23 weeks, and five patients were long-term survivors. VSLI was generally well tolerated and correlated with a low 30-day mortality rate (12%). Twenty-five patients (39%) presented at least one grade 3 treatment-related adverse event, and 19% presented at least one grade 4 treatment-related adverse event. Grade 3 peripheral neuropathy-related events were described in 23% of subjects. There was only one grade 4 peripheral neuropathy-related AE (sensory peripheral neuropathy) and no incidents of grade 4 constipation. There was no grade 3 or grade 4 nausea or vomiting [104].

### 2.3. Lymphoma

B-cell lymphoproliferative tumors are characterized by an increased proliferation of B-lymphocytes with reduction of normal hematopoiesis and infiltration of several extramedullary sites [105].

The first-line therapy commonly includes chemotherapy protocols that use several drugs. In spite of the favorable survival percentage, these multi-drug therapies present a high grade of toxicity, and a relevant rate of subjects are also refractory to the treatment or relapse. Several conditions have been reported to justify this resistance to treatment. In particular, genetic changes in certain onco suppressor genes, such as p53, might be linked to an ineffective chemotherapeutic protocol.

Monoclonal antibodies that link to CD20, an antigen which is present on the membrane of B-cells in the greater part of B-cell lymphoproliferative malignancies, are used in CD20+ lymphoma therapy. They can stimulate complement-dependent cytotoxicity (CDC) via the Fc domain and antibody-dependent cellular cytotoxicity (ADCC) via the contact to Fcγ receptors [106]. They can also be combined with conventional chemotherapy treatment. However, antibody-based immunotherapy efficacy is essentially limited by the presence of adequate quantities of tumor antigen on the cell surface [107]. Mechanisms of lymphoma immuno-resistance in vivo are not clearly understood. CD20 reduction after therapy with rituximab (an anti-CD20 antibody) has been described [108,109], therefore different therapeutic targets, such as CD19, have been suggested [110] and employed into clinical trials such as the anti-CD19 antibody denintuzumab and anti-CD19/CD3 BiTE antibody blinatumomab [111].

In any case, the usage of nanoparticles in lymphoma immunotherapy seems able to decrease the rate of relapse or refractoriness, as stated in in vitro and in in vivo pre-clinical and clinical experimentations. Moreover, these studies have allowed the elucidating of some specific mechanisms of nanoparticle effects, such as the activation of cytotoxic activity and the strengthening of immune response.

Silica-coated magnetic nanoparticles with combined ovalbumin increased the generation of cytokines and antigen uptake in BM-derived DCs [112]. Moreover, this provoked an antigen-specific cytotoxic T lymphocyte immune response and stimulated antigen-specific Th1 cell activities, including IFN-γ and IL-2 generation. Results demonstrated that the immune-stimulatory actions of silica-coated magnetic nanoparticles with conjugated ovalbumin were able to block the tumor proliferation in EG7-OVA (mouse lymphoma-expressing ovalbumin tumor-bearing mice model) [112].

Likewise, in different in vivo studies, a remarkable increase in anti-tumor efficacy and an enhanced immune response was observed for the Dox/PEG-Fmoc-NLG group compared to Doxil or the free Dox group in an A20 lymphoma mouse model. Although the treatment itself is not immunotherapy, flow cytometric analysis demonstrated that therapy increased the numbers of global and functional CD4+/CD8+ T cells with simultaneous reduction of MDSCs [113].

Wi et al. settled mannose (MN)-labeled poly(d, l-lactide-co-glycolide) (PLGA) nanoparticles (MN-PLGA-NPs) encapsulating tumor-specific antigens for targeted transport to mannose receptors (MN-R) on DCs without ex vivo manipulation [114]. The MN-PLGA-NPs demonstrated DC-specific transport in tumor-bearing mice, causing the appearance of stimulated DCs, which were transferred to lymphoid organs, provoking activation of cytotoxic CD8+ T cells. MN-PLGA-NPs demonstrated important therapeutic effectiveness in EG7 lymphoma tumor-bearing mice. Still, in the context of a non-specific enhancement of the immune response against lymphomatous pathology, a clinical trial demonstrated that nanoparticles stimulate the survival of effector T cells as well as memory T cells [115].

A different mechanism of action exerted by nanoparticles in lymphoma immunotherapy might involve cytokine production. Cytokines generated by several types of cells can increase or block immune responses against lymphoma and the use of nanoparticles could amplify the antineoplastic effect of specific cytokines. A study ascertained whether administration with OVA-NPs trapping IL-7 (OVA-NPs-IL-7) is able to cause anti-tumor immune responses in vivo [116]. Pre-treatment with a subcutaneous administration of OVA-NPs deferred the progression of a thymic lymphoma, while OVA-NPs-IL-7 inhibited the proliferation of E.G7-OVA tumor cells. The use of OVA-NPs-IL-7 increased the rate of cytotoxic T cells specific for OVA. When the tumor-free mice inoculated with OVA-NPs-IL-7 plus EG.7 cells were rechallenged with E.G7-OVA cells, they displayed a decreased proliferation with respect to that in the control mice [116]. Therefore, a single subcutaneous injection of OVA-NPs-IL-7 into animals provoked tumor-specific and also memory-like immune responses. Several other in vivo studies performed on experimental animal models confirmed these findings, showing that the use of nanoparticles determines an important increase in the immune response that could enhance the efficacy of immunotherapy.

As for the direct effects of the use of nanoparticles on the immunotherapy of lymphomas, Wu and colleagues conjugated liposomal adriamycin with Fab fragments of rituximab and demonstrated that CD20-directed liposomes can arrive to the tumor site and remarkably extend the survival of lymphoma-bearing animals [117].

In a different experimentation, biotinylated CD20 and CD3 antibodies were combined onto the surface of streptavidin-modified ultra-small Fe_3_O_4_ nanoparticles to generate a bi-specific nanoplatform (BSNP). This platform can hit CD20+ Raji cells and increase the T-cell-caused cell destroying killing in vitro. Moreover, it can also block lymphoma progression and extend the survival of a non-Hodgkin’s lymphoma (NHL) xenograft experimental model in vivo [118]. A possible mechanism of action is that, while BSNP can directly provoke the programmed cell death of Raji cell through a CD20-mediated effect, T cells are deposited around lymphoma cells by the BSNP, causing a T-cell-mediated tumor cell lysis.

A different therapeutic approach was proposed in which chemotherapy, immunotherapy, and nanoparticles were used [119]. Elevated dosages of hydroxychloroquine and chlorambucil were inserted into nanoparticles covered with an anti-CD20 antibody. These compounds were capable of destroying not only p53-mutated lymphoma cell lines presenting a small quantity of CD20, but also primary cells isolated from chronic lymphocytic leukemia (CLL) subjects. Their efficacy was also demonstrated in a model of Burkitt’s lymphoma. In vitro and in vivo data showed that the capacity of anti-CD20 nanoparticles to destroy lymphoma cells was higher with respect to free cytotoxic agents or rituximab.

In a phase-I/phase-II study, authors aimed to evaluate the safety and efficacy of nab-paclitaxel in subjects with relapsed/refractory lymphoma. Eligible subjects had to be relapsed or refractory to ≥2 prior systemic therapies. They received weekly nab-paclitaxel on days 1, 8 and 15 every 28 days. Dosing was started at 100 mg/m^2^ with dose escalations in 25 mg/m^2^ increments up to 150 mg/m^2^ in a classic 3 + 3 design. Twenty subjects (median five prior regimens) were enrolled. The maximum dose administered was well tolerated and grade 3/4 hematologic adverse events (neutropenia 25%, thrombocytopenia 20%, and anemia 15%) were acceptable. However, the overall response rate was 10% with only two partial responses, conducting to a decision to close the study prematurely [120].

A particular role of nanoparticles appears to be present in some specific forms of immunotherapy of lymphomas such as hyperthermia. The combined use of hyperthermia, immunotherapy, and nanotechnologies could remarkably increase the effectiveness of this therapeutic approach. Hyperthermia has been employed to treat a broad multiplicity of tumors [121], and it is generally employed in combination with other treatment such as immunotherapy with cytokines, interleukins, or interferons [122,123].

However, some reports stated that heat therapy increases the immunogenicity of tumor cells [124,125]. Indeed, several experiments confirmed the presence of an anti-tumor immunity provoked by hyperthermia [126] and an increased anti-tumor action of combined treatment employing hyperthermia and immunotherapy with cytokines such as interleukin-2 (IL-2) and GM-CSF [127].

As magnetite nanoparticles produce heat in an alternating magnetic field (AMF) owed to hysteresis loss, a magnetite cationic liposome (MCLs) system for intracellular hyperthermia was implemented. MCLs were settled to increase adsorption and accumulation in tumor cells [128].

Tanaka et al. evaluated the effects of hyperthermia performed in association with DC immunotherapy [129].

In an experimental animal model of EL4 T-lymphoma [129], MCLs were administered into an EL4 nodule in C57BL/6 mice, which were submitted to AMF. Hyperthermia was reiterated twice with 24 h intervals. After hyperthermia, immature DCs were inoculated into the EL4 nodule. The treatment led to a full remission of lymphoma in 75% of the animals, while the rate of remission was 12.5% in animals treated by hyperthermia alone [129]. This new treatment was termed heat immunotherapy and might be applicable to subjects with advanced lymphoma.

Similarly, encouraging outcomes, at least concerning the impact on the tumor microenvironment, have been obtained by combining photo immunotherapy [130] and nanotechnologies.

The group coordinated by Zhen employed a ferritin-nanoparticle changed with a fibroblast-activation protein-specific single-chain variable fragment (scFv) and a photosensitizer [131]. This compound can target cancer-associated fibroblasts (CAFs) and eliminate CAFs by photo-irradiation. Subsequent studies established that this CAF-eradication treatment increased diffusion of nanoparticles and other substances [132]. With respect to conventional photodynamic therapy (PDT), nanoparticles can amplify the results of immunotherapy and PDT.

The use of the new techniques could also be useful for overcoming chemoresistance. Yao et al. stated that combining rituximab, the anti-lymphoma mAb, with silver nanoparticles prevented rituximab from going into the cells and protracted drug/cell contact [133]. Moreover, nanocombined rituximab caused an increased capping of CD20 molecules on the cell membrane, thus promoting therapeutic efficiency. This indicates that the nanocarrier serves not only as a platform for drug delivery but modifies antibody performance at the molecular level.

The use of new nanotechnological approaches applied to immunotherapy could increase the possibility for a better identification and subsequent treatment of lymphoma cells. In the past, Raman spectroscopy has been employed for the detection of hematological tumor cells such as myeloma or leukemia cells [134,135].

The nanoconjugates were also employed as a Raman probe to recognize single live lymphoma cells with great specificity through surface enhanced Raman scattering (SERS) [136]. Moreover, by incorporating the single-cell level recognition specificity and sensitivity of SERS with directed and improved depletion capability, nanoconjugates can be used as an encouraging instrument in lymphoma theranostics [133].

Finally, in the future, the use of nanoparticles could be useful for enhancing the effectiveness of commonly used immunotherapy drugs. Nanoparticles can transport small-molecule drugs to block tumor-associated macrophage (TAM) activity. Bruton’s tyrosine kinase (BTK) is increased on TAMs and stimulates angiogenesis, tumor advancement, and immunosuppression [137]. Ibrutinib (IBR), an irreversible BTK inhibitor and a breakthrough drug in the treatment of leukemias and lymphomas, can reduce the support of TAMs to tumorigenesis, thus blocking the immunosuppression determined by TAMs. However, as a small molecule, IBR is quickly eliminated by the kidneys, which reduces its effectiveness. Qiu et al. implemented a nanoplatform based on amphiphilic egg phosphatidylglycerol (EPG), sialic acid (SA), and IBR (SA/IBR/EPG) [138]. With its amphiphilic EPG structure, this nanoparticle has elevated IBR-linking ability and long-lasting circulation time. After administration, the SA/IBR/EPG nanoparticle modulated anti-tumor immunity.

### 2.4. Multiple Myeloma

In spite of extraordinary current progresses in therapy employing new drugs, multiple myeloma (MM) remains a not-curable neoplasm [139,140,141,142,143].

Several data propose that the alteration of immunological response allows MM cells to escape from the immune surveillance. In fact, one of the characteristics of MM is represented by the occurrence of a tolerant BM milieu that provides a proliferation advantage to the neoplastic cells, and several compounds are presently under evaluation to target BM immune cells [144].

An interesting possible target for the implementation of an efficacious immunotherapy of MM is the B-cell maturation antigen (BCMA), a component of the tumor necrosis factor (TNF) receptor superfamily and the receptor for linking of B-cell activating factor (BAFF) and the proliferation-inducing ligand (APRIL). This molecule presents several advantages, including its expression, limited to MM cells and plasma cells, and the fact that it has an essential action in stimulating neoplastic cell proliferation and chemoresistance [145]. Presently, BCMA is being aimed at by various immunotherapeutic approaches, including mAbs, bispecific T-cell compounds, and CAR-T, with encouraging preclinical results. However, BCMA-targeted immunotherapy needs to be deeply improved before it can find effective clinical application with a satisfactory therapeutic index.

**Figure 3:**
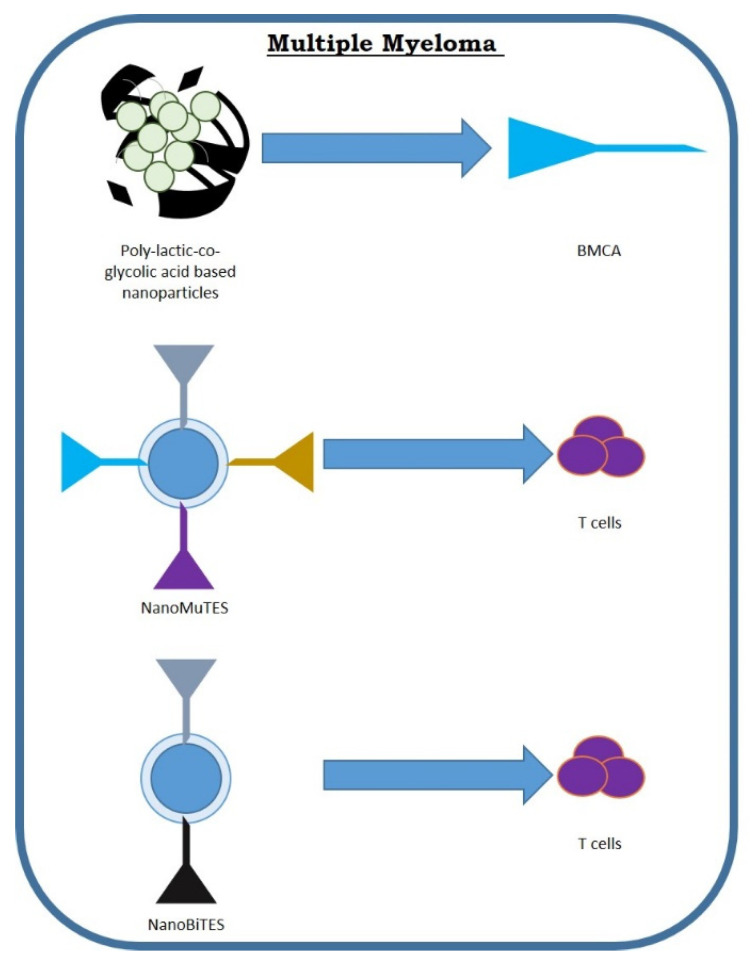
Possible targets of nanoparticles in the treatment of myeloma cells.

Bae et al. evaluated an immunogenic heteroclitic BCMA72-80 [YLMFLLRKI] peptide originated from human BCMA protein and studied its possible therapeutic use as an adoptive T-cell treatment [146]. YLMFLLRKI peptide has a powerful HLA-A2-linking affinity with enhanced immunogenicity with respect to the original BCMA72-80 peptide and provokes a strong BCMA-specific memory CD8+ CTL response against MM cells (Figure 3).

More recently, they demonstrated YLMFLLRKI-peptide-encapsuled liposomes showed increased peptide transport to DCs cells [147], while poly(lactic-co-glycolic acid (PLGA)-based nanoparticles displayed a slow increase in peptide uptake by APCs, and provoked BCMA-specific CTL with greater anti-tumor effects (CTL proliferation, CD107a degranulation, and IL-2, IFN-γ, and TNF-α generation) against primary CD138+ plasma cells and MM cell lines. The enhanced immunological effect was correlated to increased Tetramer+/CD45RO+ memory CTL, and protracted preservation of central memory (CCR7+ CD45RO+) CTL, with greater anti-MM effect [147]. These data confirm that a nanoparticle-based BCMA peptide transport system has a more specific anti-MM activity than free peptides.

Finally, regarding the use of myeloma immunotherapy using nanoparticles, Alhallak et al. evaluated the effect of nanoBiTEs, liposomes decorated with anti-CD3 mAbs aiming at T cells, and mAbs aiming at the tumor antigen [148]. They also prepared a nanoparticle that aims at several tumoral antigens by combining various mAbs against multiple tumor antigens for T-cell commitment (nanoMuTEs). NanoMuTEs and nanoBiTEs have an extended half-life, which allows once-a-week dosage instead of continuous e.v. administration. NanoMuTEs displayed a more powerful effectiveness against MM in vitro and in vivo with respect to nanoBiTEs aiming at only one cancer antigen. Contrasting nanoBiTEs, therapy with nanoMuTEs did not provoke reduction of a single antigen and blocked the onset of antigen-less tumor escape (Table 2).

## 3. Nanoparticles and Chimeric Antigen Receptor-Modified T Cells

The nanotechnological approach to immunotherapy of hematological neoplasms could finally find room in conditions such as the use of CARTs and vaccine therapy.

Chimeric antigen receptor-modified T cells (CAR-Ts) have emerged as a new modality for tumor immunotherapy due to their strong effectiveness against tumor cells. CAR-Ts are generated by transducing gene-encoding fusion proteins of tumor antigen-recognition single-chain Fv connected to the intracellular signaling domains of T-cell receptors. They are classified as first-, second-, and third-generation depending on the intracellular signaling domain number of T-cell receptors. CAR-T treatment has demonstrated to be effective for subjects with hematological malignancies, with several works reporting clinical trials about the use of CAR-modified T cells in acute lymphoblastic leukemia, chronic lymphoblastic leukemia, multiple myeloma, lymphoma, and in acute myeloid leukemia by aiming at different neoplastic antigens [149,150,151,152,153,154].

**Figure 4:**
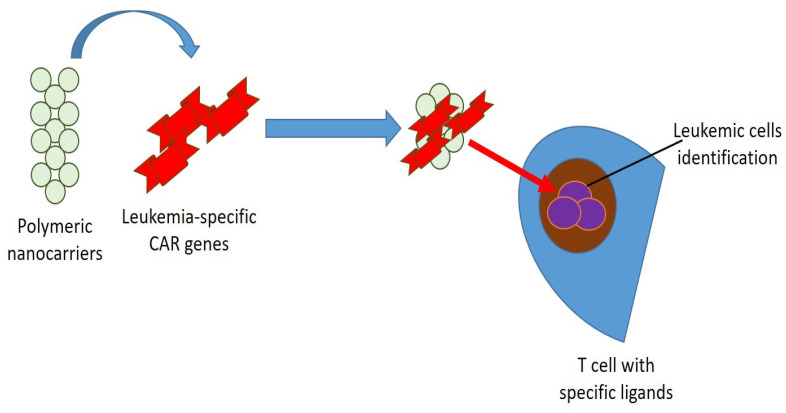
Polymeric nanocarriers can transport leukemia-specific CAR genes targeted to specific ligands on the host T cells in situ.

However, several issues are linked to this therapeutic approach, including the gene delivery [155], and a study by Smith et al. proposed the use of nanotechnology to solve some of these problems (Figure 3). They stated that polymeric nanocarriers can effectively transport leukemia-specific CAR genes targeted to specific ligands on the host T cells in situ [156]. However, several other types of nanoparticles employed for gene transport have been evaluated pre-clinically. For example, magnetic nanoparticles, such as Fe_3_O_4_, preserve constant cell transfection and plasmid transfection [157]. A different study confirmed that polymeric nanoparticles carrying DNA efficiently introduced CAR genes into T-cell nuclei and identified leukemic cells [158]. The use of nanoparticles to replace viral vectors for gene transport has confirmed advantageous and could represent a valid alternative to the procedures currently used [159] (Figure 4).

Tang et al. described an approach to “backpack” huge amounts of supporting protein drugs on T cells employing protein nanogels (NGs) that selectively discharge these cargos in response to T-cell receptor (TCR) activation. Employing NGs transporting an IL-15 superagonist complex, they reported that, relative to systemic dispensation of free cytokines, NG delivery selectively increased T cells by 16 times in tumors and allowed at least eight-fold higher doses of cytokine to be administered without toxicity. NG-backpacked CAR T cells eliminated tumors in four of five animals [160].

## 4. Tumor Vaccines and Nanoparticles

A key field of research in the area of hematological malignancies is represented by the preparation of vaccines to produce specific immune responses against blood neoplasms. Many antigens that are capable of generating T-cell responses are implicated in different systems regulating blocks of programmed cell death, demethylation, cell differentiation, and growth. Schemes of treatment include infusion of DCs or plasma cells, administration of vaccine-primed and ex vivo expanded autologous T cells, and infusion of BM-infiltrating lymphocytes. Moreover, new immunomodulatory drugs may produce a synergic effect with immunotherapies [161,162].

However, in spite of vaccines demonstrating encouraging results, clinical responses in tumor patients remains unsatisfactory. Different mechanisms of immune escape have been involved, including the generation of an immune-suppressed tumor milieu distinguished by chronic inflammation and the presence of suppressive components [163]. These humoral elements are generated not only by tumor cells, but also by Tregs, MDSCs, and suppressor macrophages and can block T-cell growth and cause T-cell functional damage or programmed cell death [164,165]. Moreover, there are difficulties with regard to initial off-target release, resulting in a reduction in vaccination efficacy. To attain the complete effectiveness of tumor vaccines, the essential prerequisite is represented by the use of adjuvants, allowing effective encapsulation of tumor antigens, a suitable transport to APCs, and an efficacious anti-tumor T-cell response. The usage of nanoparticles could enhance the effectiveness of vaccine treatment in hematological neoplasms.

Nanoparticles can effectively transport tumor antigens and adjuvants to APCs in lymph nodes, helping antigen presentation, and tumor immunotherapy employing nanoparticles could produce a durable vaccine effect and a deeper immune response than standard immunotherapy.

The effects of nanoparticles on the immune response during vaccine therapy has been broadly confirmed.

Han et al. prepared a chitosan nanoparticle (CH-NP)-based platform to bypass the ex vivo manipulation and stimulate an immune response through the active transport of polyinosinic-polycytidylic acid sodium salt (poly I:C) to aim at Toll-like receptor 3 (TLR3) in endosomes; this platform might be suitable in lymphoma treatment [166]. They built up CH-NPs encapsulating ovalbumin (OVA) as a model antigen and poly I:C as the adjuvant in an ionic complex. These CH-NPs demonstrated increased in vivo intracellular transport to the DCs with respect to controls after administration into tumor-bearing mice, and stimulated DC maturation, causing the occurrence of antigen-specific cytotoxic CD8+ T cells. Moreover, the CH-NPs displayed higher anti-tumor effectiveness in EG.7-tumor-bearing mice with respect to the control [166].

A different problem of vaccine therapy is represented by the need to preserve high local restriction of immune adjuvants to the tumor area. To this purpose, drug transport employing NPs can be utilized to improve their effectiveness due to their gradual and continuous discharge of drugs [167].

The possible advantages of NPs over free compounds are well known, and several clinical trials have been undertaken [168]. Elements such as silica NPs, metallic NPs, liposomes, or biodegradable polymers are mostly indicated for drug delivery [169,170]. Encouraging findings have been reported by other experimentations where the use of an antigen and adjuvant based on ONPs combined with the adjuvant (CpG) for tumor immunotherapy was prepared using antigens themselves as carriers. In vitro and in vivo experiments demonstrated that ONPs-CpG can stimulate a strong immune response, including T-cell stimulation, DUs maturity, and IFN-γ generation. ONPs-CpG demonstrated significant anti-tumor effects in vivo employing animal experimental models of lymphoma [171].

A different approach is represented by the attempt to associate vaccine therapy and the use of immunotherapies such as inhibitors of immunological checkpoints, with tumor vaccines produced from metal–organic-framework (MOF)-gated nanoadjuvants combined with small-dosage checkpoint blockade treatment being encouraging for hematological malignancies therapy [172,173,174,175].

Finally, Li et al. prepared a universal self-assembly route to incorporate immunology-connected big molecules into metal–organic-framework-gated mesoporous silica as a tumor vaccine. Core mesoporous silica nanoparticles, operating as an immunopotentiator, offer the space to host antigens, while the MOF defends from off-target discharge. Combined administration of MOF-gated mesoporous silica tumor vaccines with PD-1 blockade treatment causes a synergistic action that empowers anti-tumor immunity and decrease the efficacious dosage of an anti-PD-1 antibody with respect of that for PD-1 blockade monotreatment (1/10) in E.G7-OVA-tumor-bearing mice, producing long-lasting lymphoma suppression [176].

## 5. Challenges and Future Perspectives

Although extraordinary clinical effects have been attained by immunotherapy, several limitations reduce its clinical outcome, including limited response rates and severe collateral effects [177]. Nanoparticle-based methods resolve several of these complications, but some attention should be paid when employing such molecules, especially in regard to nanoparticle design. The ability of a nanomolecule to exert an effective action is determined by shape, particle size, surface charge, and hydrophobicity [178,179]. In particular, nanoparticle size is the most relevant element controlling the delivery of tumor antigens. Small nanoparticles may go out of blood vessels, while big nanoparticles can be confined in the extra cellular matrix. Medium-sized nanoparticles (~5–100 nm) remain in the circulation and are efficiently transported to the lymph nodes via lymphatic vessels [178,180]. Moreover, it is largely believed that non-spherical particles have greater blood circulation times, protracted margination effects, and greater penetration ability within tumors [181,182,183].

In the future, a fascinating field of investigation could be represented by the generation of DNA nanostructures. In fact, it is possible to build up DNA nanostructures [184,185]. DNA-founded nanoparticles are a new class of transport system for a huge assortment of bioactive elements. Plasmid DNA (pDNA) was employed as a therapeutic mAb platform in vivo by Jacobs et al. [186]. They were able to demonstrate that intramuscular and intratumoral transport of pDNA provoked a relevant anti-tumor response. DNA nanostructures can offer an efficacious drug transport for tumor treatment. DNA, being a genetic material, has great biocompatibility and small cytotoxicity, making it ideal for vectors. Lately, DNA-based nanoparticles, termed DNA nanoclews (DNA NC), have been designed that stock and transport Cas9 protein with a sgRNA for genome editing. DNA NC transported the Cas9/single guide RNA complexes to the nuclei of cells, allowing targeted gene editing [187]. Moreover, a DNA nanostructure generated from rolling circle amplification displayed great stability and easy functionalization, opening a novel area of expansion for functioning DNA nanostructures for immune-chemotherapeutic appliances [188].

Finally, the use of nanoparticles could be useful in investigating one of the last frontiers of the pathophysiology of haematological neoplasms, the extracellular vesicles (EVs). EVs are naturally generated cellular lipid bilayer particles, which transport a particular molecular content. Due to their effects in tumor pathogenesis, circulating EVs can be a relevant source of novel markers useful for tumor diagnosis and monitoring. Laurenzana et al. settled a new method based on nanoparticle tracking analysis. In clonal plasma cell malignancies, including MM, this technique allowed the recognition if specific MM EVs, and the characterization of their size, amount, and microRNA content, allowing significant distinction between MM and healthy controls [189].

## 6. Conclusions

Pre-clinical and clinical research have revealed the strong advantages of targeted nanomedicines in treating hematological malignancies [190].

The most evident advantage of nanoparticles is represented by their tunability such that they can be constructed to different sizes, shapes, and functions, making them capable of loading different drugs and attaining simultaneous transportation of different therapeutic agents. The nanosize and surface charge properties of nanodrugs are effective in improving drug delivery efficiency. The high load and rich surface modification methods of nanomaterials provide various possibilities for improving the biocompatibility and pharmacokinetics of drugs, as well as their targeting. In addition, a nanomedicine-loading platform can load multiple drugs simultaneously and design the optimal proportion of combined drug schemes, which can improve the efficacy of drugs and reduce the occurrence of drug resistance [191].

Moreover, they can accrue in neoplastic tissues much more than in normal cells, which remarkably increases the amassing of drugs in tumor cells and decreases collateral effects. Finally, nanoparticles can prevent a rapid clearance of drugs and can release drugs after specific stimuli in a spatio-temporal manner [79].

The limits of nanoparticles in tumor immunotherapy are probably to be attributed to our imperfect understanding of the immune system during the onset and the progression of hematological malignancies. The innate and adaptive immunity involves an intricate system of elements, so the impact of reduction or blockade of one element on the entire system is unsure, and the reduction of one or more elements might be balanced by an increase of other pathways. Moreover, there are several alarms about the possible risks caused by nanoparticles. The main problem is due to their possible immunogenicity. Nanoparticles themselves can be antigenic, and the immune reaction toward nanoparticles might hasten their removal, thus reducing their effectiveness, while a strong stimulation of immune system can cause the onset of critical side effects such as hemolysis and thrombogenesis [192].

Moreover, although nanomaterials are more harmless than conventional drugs, their possible side effects on human health should be evaluated as well. Nanotoxicology is a particular branch of toxicology that examines the influence of nanomaterials on living organisms and elaborates the methods to avoid such effects [193]. The dimensions of NPs cause a series of dangers, extending from subcellular and cellular planes to effects involving the whole body [92]. For instance, NPs smaller than 15–20 nm can pass the blood–brain barrier and blood–retinal barrier, which may provoke their amassing in different tissues and compromise health. Moreover, after NPs arrive at the systemic circulation, hypothetically these molecules interrelate with red or white cells, platelets, plasma proteins, and coagulation factors. Even smaller molecules could disturb with macromolecules, altering several biological processes, and even causing the stimulation of programmed cell death or necrosis [92]. Augmented levels of NPs may alter the movement and growth of cells [92,194]. Furthermore, some nanomaterials are harmful to cells due to their functional properties. Carbon-based NPs might provoke an alteration of cell membrane phospholipids, causing a structural injury and dysfunction of cells. Metal oxides are predisposed to redox reactions that generate electrons and cause further cytotoxicity.

Research about the capability of NPs as therapeutic factors still needs further evaluation and enhancement [191]. To transfer nanotechnology into nanomedicine, it is essential to know any possible toxicity caused by nanomaterials and to plan methods to decrease any damaging effects. Surface adaptation of nanomaterials to increase their compatibility and accurate control of the size and concentration of nanomaterials are essential to decrease toxicity and guarantee biosecurity.

Nano-immunotherapy has accomplished relevant results, especially in pre-clinical settings. However, the passage from pre-clinical research to clinical study is still troublesome. In fact, despite large clinical investigation, more studies are still required to efficiently combine nanomaterials with immune and chemotherapies, thus allowing the improvement of more efficacious nanomedicines with less adverse events and a valid medical applicability of this therapeutical methodology. Tumor nanomedicine continually needs to overcome several types of challenges [195]. Tumor milieu restrains the diffusion and local application of nano-drugs. Martin et al. theorized that the tumor milieu reduces the homogeneous delivery of both systemically dispensed and locally applied nanodrugs, so decreasing their effectiveness even when they store in cancer. Thus, they suggest that nanomedicines should integrate not only anti-tumor drugs but also elements that “normalize” the different constituents of the tumor microenvironment, causing an increased cancer perfusion and decreased levels of hypoxia. This action has the possibility of easing not only drug delivery but also to transform an immunosuppressed microenvironment into an immunostimulatory milieu [196].

The medical applicability of nanoparticles will also be determined by the project in which the nanomaterial is introduced and by the ability to effectively dispense various substances. Numerous characteristics of nanomedicines, such as harmonizing drug interactions, regulating the release of a drug–polymer linker, or conjugating a drug on the surface of the nanomedicine, might allow the synchronous dispensation of different compounds into single nanomedicines. These characteristics allow the control of pharmacokinetics and drug levels, thus supporting the improvement of synergistic actions. The particular clinical realization of these nanomedicine-based combination treatment methods will depend on whether these treatments should be programmed concurrently or consecutively or are contraindicated. Unfortunately, all the drugs can have severe collateral effects in specific subjects; thus, combining nanomedicines and treatment schedules including more than one agent must be cautiously planned to prevent dispensing an excessive drug load to patients.

However, the clinical use of nanoparticles should consider other elements. Nanomedicines have extraordinary value in the therapy of cancers but also have some shortcomings. Drugs depend on tumor cell markers, and this limits the medical applicability of drugs. Furthermore, phage-peptide-modified nanoparticles generally have the inconvenience of weak target binding and cannot specifically target the relevant cell tissue. Finally, the targeting ability of nanodrugs is extremely reduced and physical characteristics of NPs are seriously modified after they are adsorbed by proteins in physiological fluids in vivo to form protein coronas [197].

The transition from pre-clinical study to clinical study is still difficult. The most important point is represented by the rational design of a nano-immunotherapy platform. The effective combination of nanomaterials with existing immunotherapy strategies to regulate anti-tumor immunity provides room for clinical transformation.

Therefore, vast efforts are still required to improve the properties of nanoparticles before they can be translated to routine clinical practice. However, with deeper knowledge of tumor immunology and nanomedicine, nanoparticles will transform immunotherapy of hematological malignancies, and in the future they will reveal their great potential.

## Figures and Tables

**Table 1:** Mechanisms through which nanoparticles can enhance the efficacy of immunotherapy (www.ClinicalTrials.gov, accessed on 5 September 2021).

Mechanism	Reference(s)
Better delivery of compounds with immunotherapeutic activity; increased biodistribution and tolerability	[35,36,37,38]
Effects on tumor microenvironment	[61,62,63,70]
Effects on activation and maturation of Dendritic cells	[70,71,72,73]
Transport of adjuvant molecules	[75,76,77,78]
Enhanced tumor cell localization	[80]

**Table 2:** Effects of nanoparticles on the efficacy of immunotherapeutic treatment of hematological neoplasms.

Disease	Target and Mechanism	Compound	Ref.
Acute myeloid leukemia	Heme oxygenase 1	Lipid-polymer hybrid nanoparticle (hNP) is loaded with tin mesoporphyrin (SnMP)	[84]
	Inhibition of kinases	Poly-lactide-co-glycolide core loaded with everolimus, albumin shell loaded with MAPK/STAT5 inhibitor, conjugated with monoclonal antibody against CD33 receptor.	[89]
	Proliferation of CD8+ T cells against AML cells	Superparamagnetic iron-oxide nanoparticle core decorated with two humanized signaling proteins. HLA-A2-IgG4 hinge dimer molecules are conjugated to the core nanoparticle together with humanized anti-CD28 antibodies.	[90]
Acute lymphoblastic leukemia	Increased effect on CD19 cells (B leukemia cells)	Anti-CD19 antibody-conjugated, polyethylene glycol -biocompatibilized, and Nile Blue Raman reporter-tagged gold nanoparticles of urchin-like shape. Doxorubicin encapsulated in polymeric nanoparticles with targeting ligands against CD19.	[94] [95]
	Increased effect on CD3 cells (T leukemia cells)	Gelatin nanoparticles linked to NeutrAvidin and antibodies for the CD3 antigen. Asparaginase containing poly (lactic-co-glycolic acid)	[96] [97]
	Increased effect on CD 19 cells (T leukemia cells)	Gold-silver nanospheres conjugated with anti-CD19 monoclonal antibodies and marked with Nile-Blue-SERS-active molecules. Sgc8c aptamer (Apt)-Dau-AuNPs complex.	[101] [102]
Lymphoma	Increased production of cytokines (IFN γ, IL-2)	Silica-coated magnetic nanoparticles (MNPs@SiO_2_(RITC)) with conjugated ovalbumin.	[112]
	Increased antigen uptake	Silica-coated magnetic nanoparticles (MNPs@SiO_2_ (RITC)) with conjugated ovalbumin	[112]
	Increased antigen-specific Th1 cell activity	Silica-coated magnetic nanoparticles (MNPs@SiO_2_ (RITC)) with conjugated ovalbumin.	[112]
	Reduction of myeloid-derived suppressor cells	Dox-loaded PEG-Fmoc-NLG micelles.	[113]
	Effect on Dendritic cells	Mannose (MN)-labeled poly (d, l-lactide-co-glycolide) nanoparticles encapsulating tumor-specific antigens.	[114]
	Increased production of cytokines(IL-7)	OVA-bound nanoparticles encapsulating IL-7	[116]
	Increased CD4+/CD8+ T cells	Biotinylated CD20 and CD3 antibodies and ultra-small Fe_3_O_4_ nanoparticles with streptavidin and biotin.	[118]
	Action on cancer-associated fibroblasts	scFv-Conjugated and ZnF_16_Pc-loaded ferritin nanoparticle	[132]
	Protracted drug/cell contact	Rituxan conjugated to silver nanoparticles.	[133]
	Increased capping of CD20	Rituxan conjugated to silver nanoparticles.	[133]
Multiple Myeloma	Augment of specific memory CD0+ CTL response against MM cells	Heteroclitic BCMA_72-80_ [YLMFLLRKI] peptide-encapsulated liposome or poly (lactic-co-glycolic acid) nanoparticles.	[147]
	Effect on cytokine production	Heteroclitic BCMA_72-80_ [YLMFLLRKI] peptide-encapsulated liposome or poly (lactic-co-glycolic acid) nanoparticles.	[147]
	Blocking of tumor antigen escape	Nanoparticle-based bispecific T-cell engagers (nanoBiTEs), decorated with anti-CD3 monoclonal antibodies (mAbs) targeting T cells, and mAbs targeting the cancer antigen.	[148]
